# Research progress on diagnosing retinal vascular diseases based on artificial intelligence and fundus images

**DOI:** 10.3389/fcell.2023.1168327

**Published:** 2023-03-28

**Authors:** Yuke Ji, Yun Ji, Yunfang Liu, Ying Zhao, Liya Zhang

**Affiliations:** ^1^ The Laboratory of Artificial Intelligence and Bigdata in Ophthalmology, Affiliated Eye Hospital of Nanjing Medical University, Nanjing, China; ^2^ Affiliated Hospital of Shandong University of traditional Chinese Medicine, Jinan, Shandong, China; ^3^ Department of Ophthalmology, The First People’s Hospital of Huzhou, Huzhou, Zhejiang, China

**Keywords:** artificial intelligence, fundus images, diabetic retinopathy, hypertensive retinopathy, retinal vein occlusion, retinopathy of prematurity, age-related macular degeneration

## Abstract

As the only blood vessels that can directly be seen in the whole body, pathological changes in retinal vessels are related to the metabolic state of the whole body and many systems, which seriously affect the vision and quality of life of patients. Timely diagnosis and treatment are key to improving vision prognosis. In recent years, with the rapid development of artificial intelligence, the application of artificial intelligence in ophthalmology has become increasingly extensive and in-depth, especially in the field of retinal vascular diseases. Research study results based on artificial intelligence and fundus images are remarkable and provides a great possibility for early diagnosis and treatment. This paper reviews the recent research progress on artificial intelligence in retinal vascular diseases (including diabetic retinopathy, hypertensive retinopathy, retinal vein occlusion, retinopathy of prematurity, and age-related macular degeneration). The limitations and challenges of the research process are also discussed.

## 1 Introduction

**FIGURE 1:**
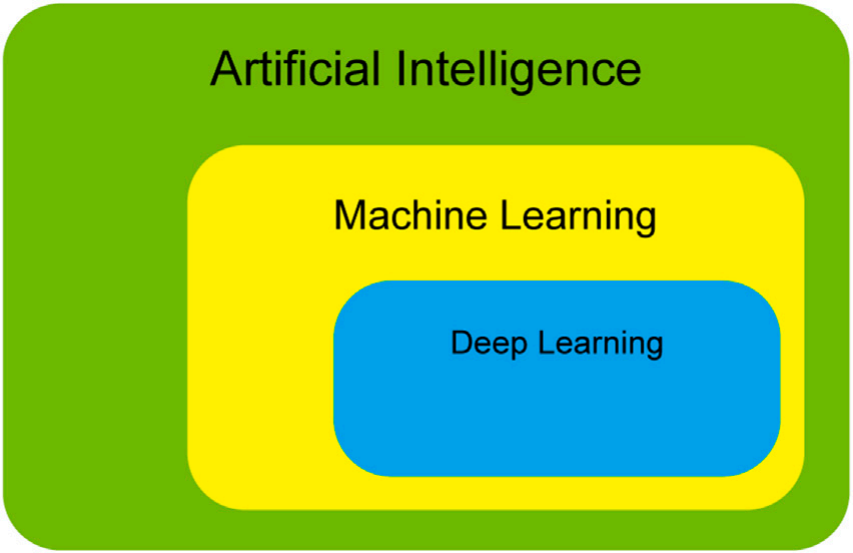
The relationship of AI, ML, and DL.

In 1956, artificial intelligence (AI) was first proposed. As a branch of computer science, the purpose of AI is to develop and study computer methods to simulate and expand human intelligence and perform complex tasks (Hamet and Tremblay, 2017). Machine learning (ML) is a subfield of AI, where machines learn and mark a large amount of measured data or features through statistical algorithms to use the generated empirical model to complete the task (Deo, 2015). ML can perform the classification task, and the classifier needs to learn to identify the tag features of the research object and then classify the task according to the tag features, which mainly depends on the resolution of the selected features. Deep learning (DL) is a subfield of machine learning, a multilayer neural network, and a machine learning method (LeCun et al., 2015). DL is powerful and can not only perform classification tasks, but also extract features. A single deep learning network can perform two tasks simultaneously, extract the features of a given classification problem, and then classify them. Compared with ML, DL has a special advantage; that is, with the increase in training data, the performance of DL will improve, whereas the performance of ML will reach saturation with the increase in data. The relationship diagrams for AI, ML, and DL are shown in Figure 1.

With the rapid development of computer science in recent years, AI has made significant progress. AI has been applied in the field of medicine, especially in ophthalmology, and the clinical application of AI is particularly extensive. AI has been used to develop AI models for automatic diagnosis, screening, classification and treatment, especially in ophthalmic diseases such as ocular surface diseases (Ji et al., 2022b), anterior segment diseases (Ting et al., 2021), cataracts (Tognetto et al., 2022), glaucoma (Coan et al., 2023), and retinal diseases (Ting et al., 2019).

Retinal vascular disease (RVD) is a major retinal disease. The vascular system of the retina is one of the components of the systemic circulatory system. There are many causes of retinal vascular diseases, including the effects of local eye diseases and systemic diseases on retinal vessels, which can be divided into the following categories: 1) retinal vascular obstructive diseases, such as retinal vein occlusion; 2) the effects of systemic diseases on retinal vessels, such as diabetes and hypertension; 3) retinal vascular inflammatory immune diseases, such as retinal periphlebitis; and 4) retinal vascular abnormalities and developmental abnormalities, such as retinopathy of prematurity. Retinal vascular disease can cause irreversible damage to retinal cells and can seriously affect the vision of patients. If patients are not treated in time, they will experience serious vision loss or blindness. Therefore, for patients with retinal vascular disease, early detection, diagnosis, and treatment are particularly important, but relatively insufficient resources for ophthalmic diagnosis and treatment greatly limit the early diagnosis and treatment of retinal vascular diseases. In recent years, AI has become increasingly used in ophthalmology, especially in image recognition and processing of retinal vascular diseases, which provides a new possibility for early diagnosis and treatment. This review summarizes the research achievements of AI for the diagnosis of retinal vascular diseases in recent years and discusses the limitations and challenges of the research.

## 2 Basic process of the medical artificial intelligence diagnosis model for research

**FIGURE 2:**
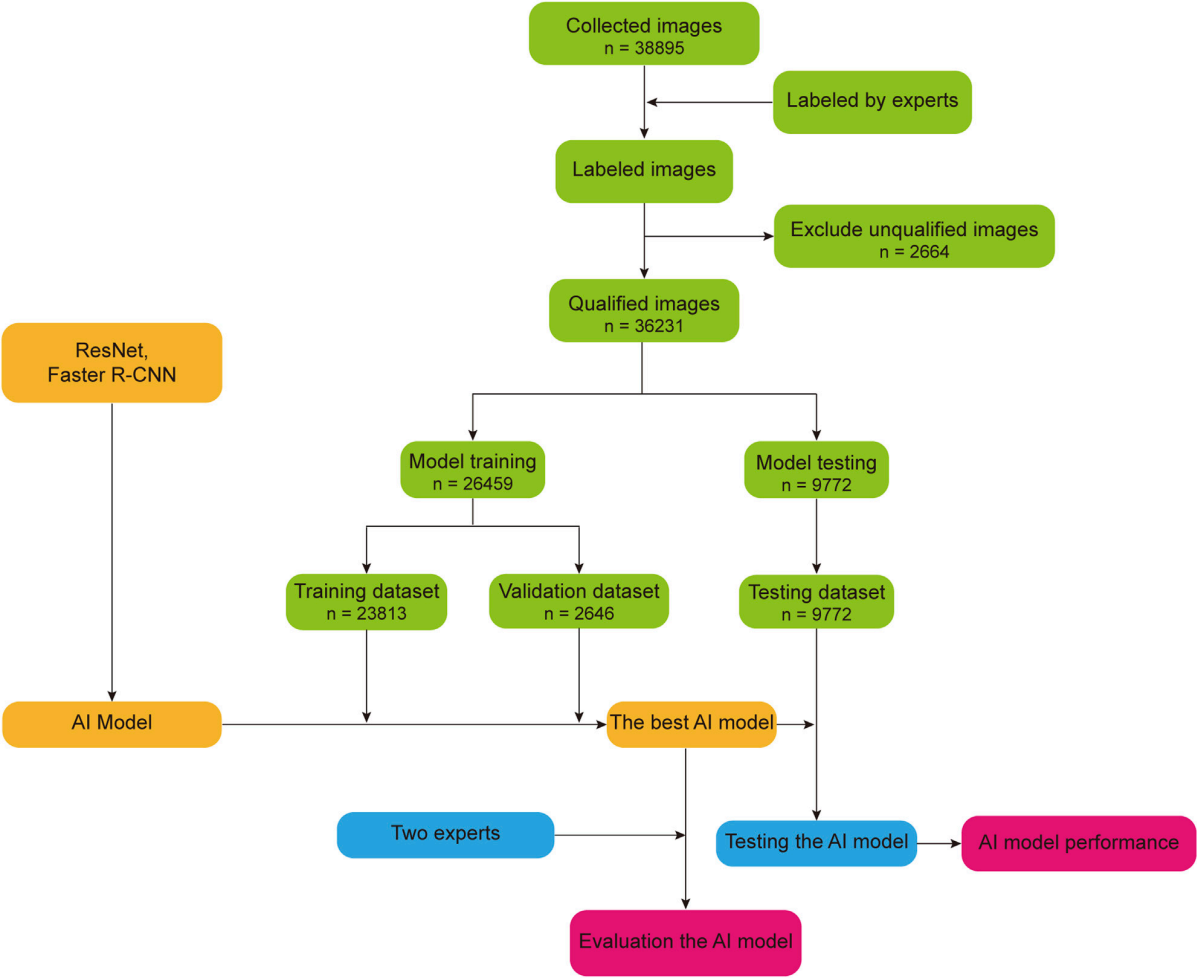
Basic flow chart of the AI diagnosis model for research.

Using the AI model by Tong et al. (2020), we drew a basic flow chart of the AI research model, as shown in Figure 2. First, the experts mark the collected images, remove the unqualified images in the labeled images, and randomly divide the remaining qualified images into a training dataset, validation dataset, and test dataset according to a certain proportion. Second, the training dataset and validation dataset are used to train and optimize the AI model to obtain the best performing AI model. Finally, we used the test dataset to test the AI model and compare the AI model’s performance with the experts.

## 3 Application of artificial intelligence in retinal vascular diseases

### 3.1 Application of artificial intelligence in diabetic retinopathy

Diabetes is a common metabolic disease that causes extensive damage to many tissues and organs in the body. Diabetic retinopathy (DR) is one of the most serious microvascular complications of diabetes and a common cause of blindness (Lim et al., 2023). The incidence of DR is primarily related to the course of diabetes and the degree of disease control. The longer the course of diabetes, the higher the incidence of DR (Huang et al., 2023). At present, the pathogenesis of DR is unclear, but glucose metabolism disorder is the root cause of DR (Han et al., 2023). In the early stage of DR, patients with general ocular symptoms can experience various visual impairments with the development of the disease, among which flash sensation and vision loss are the most common (Grauslund, 2022). Clinically, DR is divided into non-proliferative diabetic retinopathy (NPDR) and proliferative diabetic retinopathy (PDR). The most important sign of PDR is retinal neovascularization (Sheng et al., 2022). According to the severity of DR, DR is divided into six stages: stage I, microhemangioma and small hemorrhagic spot; stage II, yellow-white rigid exudation and hemorrhagic spot; stage III, white cotton velvet spot and hemorrhagic spot; stage IV, neovascularization or vitreous hemorrhage; stage V, neovascularization and fiber proliferation; and stage VI, neovascularization and fiber proliferation, accompanied by traction retinal detachment (Mehra et al., 2022; Yang et al., 2022). The treatment of DR mainly includes the following aspects: 1) strict control of blood glucose levels, which can slow the occurrence and progression of DR, 2) laser photocoagulation, 3) vitrectomy and intraocular photocoagulation, and 4) vitreous injection of anti-VEGF drugs (Li F. et al., 2022; Wang et al., 2022).

By analyzing the fundus examination images of DR patients, AI can complete the automatic diagnosis of DR, which is of great significance in improving the diagnostic and work efficiency of doctors. Li X. et al. (2022) constructed an intelligent diagnosis model for DR based on Inception-v4 to assist in the diagnosis of AI. They used 8,739 fundus images for the AI model training and evaluated them using the Messidor-2 dataset. In addition, they compared the performance of the model with that of ophthalmologists. The final results showed that the AUC, sensitivity, and specificity of the model were 0.992, 0.925, and 0.961, respectively, which were better than those of ophthalmologists. To better assist the diagnosis of severe DR, Zhang et al. (2022a) developed an AI model that can diagnose DR automatically on the basis of Inception V3 and applied The Kaggle public dataset to the development and validation of the AI model. After validation, the sensitivity, specificity, and AUC of the model for diagnosing severe DR were 0.925, 0.907, and 0.968, respectively. Zhao et al. (2022) constructed several DR prediction models using five different machine learning algorithms (Random Forest, Logistic Regression, Extreme Gradient Boosting, Support Vector Machine, K-Nearest Neighbor) and used the eye data of 7,943 patients to train and test the AI model. In addition, they compared different AI models to predict the performance of DR. After testing, the performance of the Extreme Gradient Boosting model was found to be the best, and its AUC, accuracy, sensitivity, specificity were 0.803, 0.889, 0.740, 0.811, respectively.

To build an AI model that can automatically detect DR, Hassan et al. (2022) constructed a DR detection model based on the VGG-16, ResNet-50, and U-Net. They collected 1804 fundus images, used them to train the AI model, and validated the model on external datasets. After validation, the accuracy of the model for the DR diagnosis was 0.9938. Islam et al. (2022) proposed an AI model that can detect DR based on supervised contrastive learning and used the APTOS 2019 Blindness Detection dataset and Messidor-2 dataset to train and test the AI model. After testing, the accuracy of the DR detection model was 0.9836 and the AUC was 0.9850. Using a deep learning algorithm, Elgafi et al. (2022) proposed an AI model that can detect DR using optical coherence tomography (OCT) images. In this study, 188 OCT images were collected and applied to the training and validation of the AI models. Finally, the accuracy of the model was verified to be 0.9681. By learning the characteristic lesions in the fundus images of DR patients, AI can detect DR, which can facilitate the early detection of DR patients, thereby reducing and improving clinical work pressure.

Zhang et al. (2022b) constructed a deep graph correlation network (DGCN) model through a convolution neural network, which can automatically classify DR without professional labeling. In this study, EyePACS-1 and Messidor-2 datasets were used to train and test the model. Finally, the results showed that the accuracy, sensitivity, and specificity of the model on the EyePACS-1 dataset were 0.899, 0.882, and 0.913, respectively, and the accuracy, sensitivity and specificity of the model on the Messidor-2 dataset were 0.918, 0.902, and 0.930, respectively. To assist DR classification, Zhang W. F et al. (2022) developed an AI classification model based on ResNet-34 and Inception-v3 and used 1,089 fundus images to train and test the model. After testing, the AUC of the model was 0.958 and the kappa score was 0.860. Katz et al. (2022) constructed an AI model based on W-net, which can automatically classify DR. They collected 6,981 fundus images and used them to train and test the AI model. The final results showed that the accuracy of the model was 0.989. We summarize the above research, as shown in Table 1.

**TABLE 1:** Research summary of artificial intelligence in diabetes retinopathy.

Year	Country or region	Authors	Task	Dataset (disease images)	AI algorithm	Output
2021	China	Li et al. (2022)	Diagnosis	8,739 images, Messidor-2 dataset (8,379 images)	Inception-v4	AUC = 0.992, Sensitivity = 0.925, Specificity = 0.961
2022	China	Zhang et al. (2022a)	Diagnosis	The Kaggle public dataset (4,192 images)	Inception V3	Sensitivity = 0.925, Specificity = 0.907, AUC = 0.968
2022	China	Zhao et al. (2022)	Diagnosis	7,943 patients’ data (1,692 images)	Random Forest, Extreme Gradient Boosting, Logistic Regression, Support Vector Machine and K-Nearest Neighbor	AUC = 0.803, Accuracy = 0.889, Sensitivity = 0.740, Specificity = 0.811
2022	America	Hassan et al. (2022)	Detection	1804 images (920 images)	VGG-16, ResNet-50, U-Net	Accuracy = 0.9938
2022	Bangladesh	Islam et al. (2022)	Detection	APTOS 2019 Blindness Detection dataset, Messidor-2 dataset (5068 images)	Supervised competitive learning	Accuracy = 0.9836, AUC = 0.9850
2022	Egypt	Elgafi et al. (2022)	Detection	188 images (88 images)	Deep learning	Accuracy = 0.9681
2022	China	Zhang et al. (2022b)	Grading	EyePACS-1, Messidor-2 (5849 images)	Deep graph correlation network	EyePACS-1: Accuracy = 0.899, Sensitivity = 0.882, Specificity = 0.913
						Messidor-2: Accuracy = 0.918, Sensitivity = 0.902, Specificity = 0.930
2022	China	Zhang F et al. (2022)	Grading	1,089 images (1,089 images)	ResNet-34, Inception v3	AUC = 0.958, Kappa = 0.860
2021	Israel	Katz et al. (2022)	Grading	6,981 images (6,981 images)	W-net	Accuracy = 0.989

### 3.2 Application of artificial intelligence in hypertensive retinopathy

Hypertensive retinopathy (HR) is a common retinal vascular disease caused by long-term hypertension (Ji et al., 2022a). Fundus changes in HR patients are related to age and disease course. The older the age of HR patients, the longer the course of the disease and the higher the incidence of fundus lesions (Cheung et al., 2022). In the early stage, there is often no obvious change in the fundus of HR patients. With the progression of the disease, the retinal artery gradually changes organically, and the wall of the retina begins to harden, appearing as a copper wire or silver wire (Di Marco et al., 2022). The diameter of the artery gradually narrows, and the proportion of arteries and veins gradually decreases (Dziedziak et al., 2022). Retinal hemorrhage, hard exudation, cotton velvet spots, and other changes occur in the fundus; and optic disc edema may occur in severe cases (Liu et al., 2021; Badawi et al., 2022). According to the progression and severity of the disease, HR is divided into four grades: grade I, vasoconstriction and narrowing; grade II, arteriosclerosis; grade III, exudation, cotton velvet spots, hemorrhage, and extensive microvascular changes; and grade IV grade III changes and optic disc edema (Wong and Mitchell, 2004; Tsukikawa and Stacey, 2020). In clinical treatment, lowering blood pressure is the most fundamental means to prevent and treat fundus changes. After the effective control of blood pressure, optic disc edema, retinal edema, hemorrhage, and exudation can be absorbed and eliminated (Klig, 2008; Del Pinto et al., 2022). If HR patients have complications such as macular edema, treatment such as intravitreal injection of anti-VEGF drugs can significantly improve their vision (Padhy and Kumar, 2018).

In many studies, AI has been used to screen and diagnose HR, and the AI model constructed in this study showed good screening and diagnostic performance and has the potential for clinical application. Han et al. (2021) constructed an AI model to screen for HR and other common eye diseases based on an anomaly detection algorithm. In this study, 90,499 fundus photos were collected and randomly divided into training, validation, and testing dataset according to a certain proportion, which were used to develop and evaluate the AI model. After testing, the AUC, accuracy, sensitivity, and specificity of the HR diagnosis model were 0.895, 0.8237, 0.8129, and 0.8275, respectively. To assist clinicians in screening HR, Arsalan et al. (2021) constructed an AI screening model using a dual-stream fusion network (DSF-Net) and a dual-stream aggregation network (DSA-Net). They evaluated the performance of the model using the DRIVE, STARE, and CHASE-DB1 dataset. After testing, the accuracy, sensitivity, specificity, and AUC value for DRIVE were 0.9693, 0.8268, 0.9830, and 0.9842, respectively; for CHASE-DB1 they were, 0.9725, 0.8222, 0.9838, and 0.9815, respectively; and for STARE they were 0.9700, 0.8607, 0.9800, and 0.9865, respectively. Arsalan et al. (2019) developed a dual-residual-stream-based vessel segmentation network (Vess-Net) model on the basis of convolutional neural networks, which is used to assist HR diagnosis and to train and test on the open datasets of DRIVE, CHASE-DB1, and STARE. Finally, the results showed that the sensitivity, specificity, AUC, and accuracy of the model for diagnosing HR were 0.8526, 0.9791, 0.9883, and 0.9697, respectively. Dong et al. (2022) collected 120,002 fundus photos and used a convolutional neural network to create a retinal AI diagnosis system (RAIDS) for the diagnosis of 10 types of retinal diseases, including HR. They randomly divided 120,002 fundus photos into training, test, and validation datasets and used them in the training and validation of the system. The accuracy of the system in identifying HR was verified to be 0.837.

AI is also used in the classification and grading of HR, which is expected to be used clinically to reduce the pressure on doctors. Abbas et al. (2021) constructed a HYPER-RETINO system based on the DenseNet algorithm to assist in the classification of HR. They collected 1,400 fundus photos and used them for the development and testing of the system. The sensitivity, specificity, accuracy, Matthews correlation coefficient, and AUC of the system were 0.905, 0.915, 0.926, 0.61, and 0.915, respectively. Akbar et al. (2018) constructed an AI model using a DL algorithm (support vector machine and radial basis function) to assist in screening and grading of HR. The INSPIRE-AVR, VICAVR, STARE, and AVRDB datasets were used to develop, train and test the model. After testing, it was found that the accuracies of the first part of the model on the INSPIRE-AVR, VICAVR, and AVRDB dataset were 0.9510, 0.9564, and 0.9809, respectively, and the accuracies of the second part on the STARE and AVRDB dataset were 0.9593 and 0.9750, respectively. We summarize the above research, as shown in Table 2.

**TABLE 2:** Research summary of artificial intelligence in hypertensive retinopathy.

Year	Country or region	Authors	Task	Dataset (disease images)	AI algorithm	Output
2021	China	Han et al. (2021)	Screening	90,499 images (26,148 images)	Anonymous detection	AUC = 0.895, Accuracy = 0.8237, Sensitivity = 0.8129, Specificity = 0.8275
2022	Korea	Arsalan et al. (2021)	Screening	DRIVE, START, CHASE-DB1 (2051 images)	Dual-stream fusion network, Dual-stream aggregation network	DRIVE: Accuracy = 0.9693, Sensitivity = 0.8268, Specificity = 0.9830, AUC = 0.9842
						CHASE-DB1: Accuracy = 0.9725, Sensitivity = 0.8222, Specificity = 0.9838, AUC = 0.9815
						START: Accuracy = 0.9700, Sensitivity = 0.8607, Specificity = 0.9800, AUC = 0.9865
2019	Korea	Arsalan et al. (2019)	Diagnosis	DRIVE, CHASE-DB1, STARE (1960 images)	Convolutional neural networks	Sensitivity = 0.8526, Specificity = 0.9791, Accuracy = 0.9883, AUC = 0.9697
2022	China	Dong et al. (2022)	Diagnosis	120,002 images (8,198 images)	Convolutional neural network	Accuracy = 0.837
2021	Saudi Arabia	Abbas et al. (2021)	Classification	1,400 images (1,000 images)	DenseNet	Sensitivity = 0.905, Specificity = 0.915, Accuracy = 0.926, Matthews correlation coefficient = 0.61, F1-score = 0.92, AUC = 0.915
2017	Pakistan	Akbar et al. (2018)	Classification	INSPIRE-AVR, VICAVR, STARE, and AVRDB (198 images)	Support vector machine, Radial basis function	Accuracy: INSPIRE-AVR = 0.9510, VICAVR = 0.9564, AVRDB = 0.9809, STARE = 0.9593, AVRDB = 0.9750

### 3.3 Application of artificial intelligence in retinal vein occlusion

Retinal vein occlusion (RVO) is one of most common retinal vascular disease, second only to diabetic retinopathy, and more common in older patients (Ren et al., 2022). The pathogenesis of RVO is related to many factors such as vascular endothelial damage, hemodynamic changes, intraocular pressure, and ocular local compression (Terao et al., 2022; Trovato Battagliola et al., 2022). In addition, the disease is closely related to arteriosclerosis, cardiovascular and cerebrovascular diseases, hypertension, diabetes, and other risk factors (Orskov et al., 2022; Tang et al., 2022). According to the location of vein occlusion, RVO is mainly divided into central retinal vein occlusion (CRVO) and branch retinal vein occlusion (BRVO), of which branch occlusion is the most common (Miao et al., 2022). In the early stage, the symptoms are characterized by a sudden loss of vision to varying degrees; mild patients may have no symptoms or only a little shadow (Pur et al., 2023), and with the progression of the disease, RVO patients have serious visual impairment (Zhang X. T et al., 2022; Sood et al., 2022). Typical fundus changes in RVO patients include retinal hemorrhage, tortuous retinal vein dilatation, extensive retinal capillary non-perfusion area, and macular edema (Irgat and Ozcura, 2023). Late patients may have complications such as vitreous hemorrhage, traction retinal detachment, and neovascular glaucoma, resulting in severe visual acuity loss and even blindness (Altintas and Ilhan, 2023; Patil et al., 2023). Some commonly used treatment methods in ophthalmology are mainly used to prevent and treat complications such as laser photocoagulation, vitrectomy, vitreous injection of hormones, or anti-VEGF drugs (Ghanchi et al., 2022; Yin et al., 2022).

As an important clinical assistant tool, AI has been widely used in the early screening of retinal vein occlusion, and especially in areas where lacking medical resources, AI can play an important role. To assist in screening for retinal vein occlusion, Chen J. S et al. (2021) constructed an AI screening model using four DL algorithms (ResNet-50, Inception-v3, DenseNet-121, SE-ReNeXt-50). They collected 8,600 color fundus photos and randomly divided them into training, validation, and test dataset according to a certain proportion for the development and testing of AI models. After testing, the Inception-v3 model’s performance was the best, and its sensitivity, specificity, F1 score, and AUC were 0.93, 0.99, 0.95, and 0.99, respectively. Nagasato et al. (2019a) constructed two AI models using the VGG-16 and support vector machine algorithms to detect branch retinal vein occlusion. They collected 465 ultrawide-field fundus images for training and validation of AI models and compared the performance of the two models. The final results showed that the detection performance of the VGG-16 model was better than that of support vector machine model, with a sensitivity of 0.940, a specificity of 0.970, and an AUC of 0.976. Nagasato et al. (2018) constructed two screening models for CRVO based on the VGG-16 and support vector machine algorithms. In this study, 363 ultrawide-field fundus images were used to develop and test AI models, and the screening performance of the two AI models was compared. The VGG-16 model had the best screening performance, with a sensitivity of 0.984, specificity of 0.979, and AUC of 0.989. Anitha et al. (2012) constructed an AI diagnosis model based on artificial neural networks to assist in the diagnosis of four retinal diseases, including central retinal vein occlusions. They collected 420 digital retinal images to send and verify their model. The results showed that the model’s accuracy, sensitivity, and specificity were 0.977, 0.960, and 0.980, respectively. To assist in the diagnosis of retinal vein occlusion, Kang et al. (2021) developed an AI diagnosis model based on a convolution neural network, and used the examination data of 2,992 eyes to develop and train the model. After testing, the AUC of this model for BRVO was 0.959 and that of CRVO was 0.988. Abitbol et al. (2022) collected 224 ultra-widefield color fundus images and constructed an AI model based on the DenseNet121 network to assist diagnose three types of retinal vascular diseases such as retinal vein occlusion. Finally, the accuracy of the model in the diagnosis of RVO was 0.884, and the AUC was 0.912.

Xu et al. (2022) constructed an AI model based on ResNet18 to assist in the classification of RVO. In their study, 501 fundus images were collected for the development and testing of the model. After testing, the classification accuracy of the model was greater than 0.97, the sensitivity was greater than 0.95, the sensitivity was greater than 0.97, and the F1 score was greater than 0.97. Zhang X. et al. (2022) constructed a VGG-CAM network model based on convolutional neural networks to assist in the diagnosis and classification of RVO. They used a local image database to train and test the model and compared it with Resnet-34, Inception-V3, and MobileNet network models. After testing, the sensitivity, specificity, Kappa coefficient, and AUC of the model for diagnosing central RVO were 0.99, 0.96, 0.88, and 0.99, respectively, and the sensitivity, specificity, Kappa coefficient, and AUC for diagnosing branch RVO were 0.94, 0.99, 0.97, and 0.99, respectively. In addition, its diagnostic performance was superior to that of other network models. It can be seen that in the clinical classification of retinal vein occlusion, compared with manual classification, automatic classification has lower cost and higher efficiency and can play an important role in clinical practice.

In addition, AI can help clinicians diagnose RVO by identifying and segmenting the characteristic lesions in the images of patients with RVO, thus reducing the workload of clinicians. Tang et al. (2021) constructed an AI model using CE-Net to help segment the non-perfusion area of the retina caused by RVO, thus helping to evaluate RVO severity. They collected 177 fluorescein angiography images for training and testing the AI model and enhanced the performance of the AI model through an adaptive histogram-based data augmentation method. After testing, the accuracy of the model was 0.883. To detect the non-perfusion area caused by RVO in optical coherence tomography angiography (OCTA) images to help diagnose RVO, Nagasato et al. (2019b) constructed an AI model based on VGG-16 and support vector machine and collected 322 OCTA images for AI model training and testing. In addition, they compared the performance of the AI model with the diagnostic abilities of seven ophthalmologists. After testing, the performance of the VGG-16 model was better than support vector machine model and the seven ophthalmologists, and its AUC, sensitivity, and specificity were 0.986, 0.937, and 0.973, respectively. We summarize the above research, as shown in Table 3.

**TABLE 3:** Research summary of artificial intelligence in retinal vein occlusion.

Year	Country or region	Authors	Task	Dataset (disease images)	AI algorithm	Output
2021	China	Chen S et al. (2021)	Screening	8,600 images (440 images)	ResNet-50, Inception-v3, DenseNet-121, SE-ReNeXt-50	Sensitivity = 0.93, Specificity = 0.99, F1 = 0.95, AUC = 0.99
2018	Japan	Nagasato et al. (2019a)	Detection	465 images (125 images)	VGG-16, Support vector machine	Sensitivity = 0.940, Specificity = 0.970, AUC = 0.976
2018	Japan	Nagasato et al. (2018)	Screening	363 images (237 images)	VGG-16, Support vector machine	Sensitivity = 0.984, Specificity = 0.979, AUC = 0.989
2011	India	Anitha et al. (2012)	Diagnosis	420 images (95 images)	Artificial neural networks	Accuracy = 0.977, Sensitivity = 0.960, Specificity = 0.980
2021	Taiwan	Kang et al. (2021)	Diagnosis	2,992 eyes (325 eyes)	Convolution neural network	AUC of branch retinal vein occlusion = 0.959; AUC of central retinal vein occlusion = 0.988
2022	France	Abitbol et al. (2022)	Diagnosis	224 images (169 images)	DenseNet121	Accuracy = 0.884, AUC = 0.912
2022	China	Xu et al. (2022)	Classification	501 images (242 images)	ResNet18	Accuracy>0.97
						Sensitivity>0.95, F1 score>0.97
2022	China	Zhang et al. (2022a)	Classification	Local image database (Not specified)	Convolutional neural networks	Sensitivity = 0.99, Specificity = 0.96, Kappa coefficient = 0.88, AUC = 0.99
2020	China	Tang et al. (2021)	Division	177 images (177 images)	CE-Net	Accuracy = 0.883
2019	Japan	Nagasato et al	Detection	322 images (128 images)	VGG-16, Support vector machine	AUC = 0.986, Sensitivity = 0.937, Specificity = 0.973

### 3.4 Application of artificial intelligence in retinopathy of prematurity

Retinopathy of prematurity (ROP), also called retrolental fibroplasia, is a proliferative retinopathy of immature or low birth weight infants (Campbell et al., 2022). Most of the infants were premature with less than 34 weeks of pregnancy, birth weight less than 1,500 g, history of inhalation of high concentrations of oxygen, or stunted low birth weight infants (Sabri et al., 2022). Preterm birth, low birth weight, and inhalation of high concentrations of oxygen are high-risk factors for ROP (Ramanathan et al., 2022). The clinical manifestations of children with ROP vary according to the course of the disease, which is divided into three areas according to the location of the lesion: area Ⅰ, a circular area with a radius of 2 times the distance from the optic disc to the fovea of the macula (Bai et al., 2022); area Ⅱ, a circular area centered on the optic disc to the sawtooth margin of the nasal side (Eilts et al., 2023); and area Ⅲ, the area excluding areas I and II (Nisha et al., 2023). According to the severity of the lesion, it was divided into five stages: stage 1, dividing line stage; stage 2, critical stage; stage 3, increment stage; stage 4, subpanretinal detachment stage; and stage 5, panretinal detachment stage (Gensure et al., 2020). For treatment, stage 1 and stage 2 can disappear naturally, so they should be observed closely (Scruggs et al., 2020); stage 3 should be treated with condensation or photocoagulation to prevent neovascularization (Barrero-Castillero et al., 2020); and stage 4 and stage 5 can be treated with a vitrectomy to remove proliferated fibrovascular tissue. Photocoagulation was performed simultaneously (Morya et al., 2022). Once ROP occurs, it progresses rapidly, and the curative effect in advanced cases is limited; therefore, it is important for children with ROP to be detected and treated early to avoid serious consequences.

To automatically diagnose ROP, Brown et al. (2018) constructed a diagnostic model based on U-Net and Inception version 1, and 5,511 retinal images were used to develop and train the AI model. In addition, they compared the AI model’s diagnostic performance with that of eight experts. The final results showed that the sensitivity, specificity, and accuracy of the AI diagnosis model were 0.93, 0.94, 0.91 respectively, whereas the average accuracy of the eight experts was 0.82. This shows that the diagnostic performance of the AI model is superior. Chen Q. et al. (2021) proposed an AI model on the basis of convolution neural network, which tcan assist the staging diagnosis of ROP. They collected 10,894 fundus images and divided them into training and testing dataset. After testing, the AUROC of the model was 0.99, the AUPRC was 0.98, and the sensitivity was 0.94. Mao et al. (2020) established an AI model that can assist in the diagnosis of ROP based on U-Net and Dense Net and analyzed the progress of ROP. They used 3,311 fundus images to train and varify the AI model. Finally, the results showed that the diagnostic specificity of the model was 0.978, the sensitivity was 0.951, and the sensitivity and specificity for the diagnosis of disease deterioration were 0.924 and 0.974, respectively. Peng et al. (2022) constructed an ADS-Net model based on DenseNet121 to assist doctors in the diagnosis of ROP. In this study, 8,733 fundus images were collected from two datasets for training and verifying the model. After validation, the accuracy of the model for diagnosing ROP was 0.9776, recall was 0.9714, precision was 0.9835, F1-score was 0.9774, and the kappa coefficient was 0.9552. Based on the above AI research results, it can be found that AI model shows superior performance in automatic diagnosis of ROP by recognizing ophthalmic examination data such as fundus images, and has the potential to be used in clinical diagnosis and treatment, which can greatly improve the work efficiency of clinicians and reduce the work pressure of clinicians.

In recent years, AI model has made a lot of research achievements in assisting the clinical staging and grading diagnosis of ROP. In order to assist in the grading and staging of ROP, Tong et al. (2020) constructed an AI model based on ResNet and faster region-based convolutional neural network (Faster-RCNN). In this study, 36,231 retinal images were collected and randomly divided into training, validation, and testing datasets. In addition, they compared the classification performance of the AI model with two retinal experts. The final results showed that, in terms of ROP classification, the accuracy, sensitivity, specificity, and F1 scores of the model were 0.903, 0.778, 0.932, and 0.761, respectively, which were better than the two retinal experts. In terms of ROP staging, the diagnostic accuracies of stages 1, 2, 3, 4, and 5 were 0.876, 0.942, 0.968, 0.998, and 0.999, respectively. Peng et al. (2021) used ResNet18, DenseNet121, and EfficientNetB2 to create an AI model for ROP staging and used 635 retinal images to train and verify the model. After validation, the recall of the model was 0.905, precision was 0.9092, the F1 score was 0.9043, accuracy was 0.9827, and Kappa was 0.9786. To detect early ROP and staging, Huang et al. (2021) constructed an ROP staging model using a through convolution neural network. They randomly divided 11,372 fundus images into training and test datasets and used them to train and test the AI model. The results showed that the accuracy, sensitivity, and specificity of the model were 0.9223, 0.9614, and 0.9595, respectively. The sensitivity and specificity of stage 1 ROP were 0.9182 and 0.9450, respectively; the sensitivity and specificity of stage 2 ROP were 0.8981 and 0.9899, respectively. Li F. et al. (2022) developed an AI model based on U-Net and Dense Net to assist in the diagnosis of children with early ROP in stage 1–3. They collected 18,827 retinal images for training and validation dataset. After validation, the sensitivity and specificity of the model were 0.9593 and 0.9929 for normal images, 0.9021 and 0.9767 for stage 1 ROP, 0.9275 and 0.9874 for stage 2 ROP, 0.9184 and 0.9929 for stage 3 ROP, respectively. AI model has made many achievements in the clinical staging and grading diagnosis of ROP. AI model can help clinicians to grade and stage diagnosis of ROP, which is more conducive to the early diagnosis and treatment of ROP patients.

To detect the blood vessels in areas I, II, and III of children with ROP and to assist in assessing the severity of ROP, Agrawal et al. (2021) built an AI model by combining U-Net and Circle Hough Transform. They collected 4,250 fundus images to develop and test the AI model, all of which were labeled by ROP experts. After testing, the model’s accuracy was 0.98. To predict the occurrence and evaluate the severity of ROP, Wu et al. (2022) constructed an AI prediction model and AI evaluation model based on OC-Net and SE-Net. They collected 7,796 retinal images for training and validation dataset. The results showed that the AUC, accuracy, sensitivity, and specificity of the OC-Net prediction model were 0.94, 0.333, 1.00, and 0.075, respectively. The AUC, accuracy, sensitivity, and specificity of the OC-Net prediction model were 0.88, 0.560, 1.00, and 0.353, respectively. We summarize the above research, as shown in Table 4.

**TABLE 4:** Research summary of artificial intelligence in retinopathy of prematurity.

Year	Country or region	Authors	Task	Dataset (disease images)	AI algorithm	Output
2018	America	Brown et al. (2018)	Diagnosis	5,511 images (977 images)	U-Net, Inception version 1	Sensitivity = 0.93, Specificity = 0.94, Accuracy = 0.91
2020	America	Chen Q. et al. (2021)	Diagnosis	10,894 images (1945 images)	Convolution neural network	AUROC = 0.99, AUPRC = 0.98, Sensitivity = 0.94
2020	China	Mao et al. (2020)	Diagnosis	3,311 images (1,393 images)	U-Net, Dense Net	Specificity = 0.978, Sensitivity = 0.951
2022	China	Peng et al. (2022)	Diagnosis	8,733 images (3,684 images)	DenseNet121	Accuracy = 0.9776, Recall = 0.9714, Precision = 0.9835, F1-score = 0.9774, Kappa = 0.9552
2020	China	Tong et al. (2020)	Classification	36,231 images (36,231 images)	ResNet, Faster region-based convolutional neural network	Accuracy = 0.903, Sensitivity = 0.778, Specificity = 0.932, F1 score = 0.761
2021	China	Peng et al. (2021)	Classification	635 images (332 images)	ResNet18, DenseNet121, EfficientNetB2	Recall = 0.9055, Precision = 0.9092, F1 score = 0.9043, Accuracy = 0.9827, Kappa = 0.9786
2020	Taiwan	Huang et al. (2021)	Classification	11,372 images (1,279 images)	Convolution neural network	Accuracy = 0.9223, Sensitivity = 0.9614, Specificity = 0.9595, Sensitivity and Specificity of stage 1 ROP = 0.9182, 0.9450, Sensitivity and Specificity of stage 2 ROP = 0.8981,0.9899
2022	China	Li and Liu (2022)	Classification	18,827 images (3,869 images)	U-Net, Dense Net	Sensitivity of diagnosing = 0.9593, Specificity of diagnosing = 0.9929, Sensitivity and Specificity of stage 1 ROP = 0.9021, 0.9767, Sensitivity and Specificity of stage 2 ROP = 0.9275,0.9874, Sensitivity and Specificity of stage 3 ROP = 0.9184,0.9929
2021	India	Agrawal et al. (2021)	Evaluation	4,250 images (2,350 images)	U-Net, Circle Hough Transform	Accuracy = 0.98
2022	China	Wu et al. (2022)	Evaluation	7,796 images (1984 images)	OC-Net, SE-Net	AUC, Accuracy, Sensitivity and Specificity of OC-Net = 0.94,0.333,1.00, and 0.075, respectively
						AUC, Accuracy, Sensitivity and Specificity of SE-Net = 0.88, 0.560, 1.00, and 0.353, respectively

### 3.5 Application of artificial intelligence in age-related macular degeneration

Age-related macular degeneration (AMD), also known as senile macular degeneration, is common in Europe, the United States, and other developed countries and is the main cause of blindness in the elderly in developed countries. Its incidence increases with age (Thomas et al., 2021). At present, the etiology and pathogenesis of AMD are not clear, and the related risk factors include age, sex, race, heredity, smoking, malnutrition, metabolic disorders, and retinal light damage (Lombardo et al., 2022; Tao et al., 2023). Most patients with AMD are more than 50 years old, have both eyes effected at the same time or successively, and have progressive visual impairment. According to clinical manifestations and pathological changes, AMD can be divided into two types: atrophic or non-exudative or dry; exudative or neovascularization or wet (Gale et al., 2023). The main feature of atrophic AMD is progressive RPE atrophy, the main changes of the fundus are vitreous warts and RPE degeneration and atrophy (Zhang et al., 2023), and the characteristic changes of exudative AMD are neovascularization under the RPE, subretinal neovascular membrane, and subretinal hemorrhage (Liberski et al., 2022; Cao et al., 2023). For treatment, because the etiology of AMD is not clear, there is still no specific drug treatment or fundamental effective preventive measures; vitreous injection of anti-VEGF drugs is mainly used for neovascular AMD (Fabre et al., 2022; Galindo-Camacho et al., 2022).

To assist clinicians in diagnosing age-related macular degeneration and distinguishing its different types, AI has carried out a lot of research in this area, with remarkable results. Han et al. (2022) collected 4,749 spectral domain optical coherence tomography (SD-OCT) images and constructed an AI model that can diagnose neovascular age-related macular degeneration using three convolution neural networks (VGG-16, VGG-19, and ResNet). They randomly divided 4,749 images into training and test datasets and used them to develop and verify the model. In addition, they compared the diagnostic performance of the model with that of ophthalmologists. The results showed that the accuracy of the model was 0.874, which was similar to that of ophthalmologists. To distinguish between different types of AMD, Tak et al. (2021) constructed a model based on convolutional neural networks and used 420 Optos wide-field retinal images for training and validation. The classification accuracy of the model was found to be 0.88. Chou et al. (2021) constructed a DL model based on EfficientNet-B3 for the differential diagnosis of neovascular age-related macular degeneration. They collected 699 fundus photographs for training and testing the model. After testing, the model showed good performance with accuracy, sensitivity, specificity, and AUC values of 0.8367, 0.8076, 0.8472, and 0.8857, respectively. Heo et al. (2020) constructed an AI model using the VGG16 model to identify the different types of AMD. In this study, 399 fundus images were used to train and verify the model, and the discrimination performance of the model was compared with that of residents. The accuracy of the model was better than that of the residents, with an accuracy of 0.9086.

In addition to extensive research on the diagnosis and classification of AMD, AI has been used to predict the severity, disease progression, and therapeutic effect in patients with age-related macular degeneration. Ganjdanesh et al. (2022) created a new DL model (LONGL-Net) based on ResNet-18 to predict the severity and progression of patients with age-related macular degeneration. They collected approximately 30,000 color fundus photographs for training and verifying the model. The average accuracy of the model was 0.905, and the AUC was 0.762. Song et al. (2022) constructed an AI model that predicted neovascular ANM based on a classified convolution neural network and a complete convolutional neural network algorithm. In total, 671 SD-OCT images were used to train and test the model. The average accuracy of the model was 0.930, the Dice coefficient was 0.873, the sensitivity was 0.873, and the specificity was 0.922. To predict the treatment effect and disease progression in patients with neovascular AMD, Yeh et al. (2022) built an AI prediction model using a new type of deep convolution neural network (Heterogeneous Data Fusion Net). They collected eye SD-OCT images from 698 patients and used them to train and test the model. In addition, they compared the predictive performance of the model with those of the ResNet50 and AlexNet models. The prediction performance of the model was better than that of ResNet50 and AlexNet, with an AUC value of 0.989, accuracy of 0.936, sensitivity of 0.933, and specificity of 0.938. Yan et al. (2020) developed an AI model based on convolutional neural networks to predict the disease progression in patients with AMD. They collected 31,262 eye OCT images and 52 related mutations. After testing, the AUC value of the model for predicting the disease progression was 0.85.

Holomcik et al. (2022) constructed an AI model on U-Net to automatically segment lesions in fluorescein angiography images of patients with neovascular AMD. They collected 9,268 images to develop and test the model. After testing, the F1 score, accuracy, and recall of the segmented lesion size were 0.65, 0.75, and 0.72, respectively, and the F1 scores, accuracy, and recall of the leakage area were 0.73, 0.80, and 0.78, respectively. He et al. (2022) created a DL model that can detect age-related macular degeneration through the ResNet-50 model and local outlier factor (LOF) algorithm and used the UCSD dataset and Duke dataset to train and test the model. Finally, the accuracy of the model was 0.9987 for the UCSD dataset and 0.9756 for the Duke dataset. We summarize the above research, as shown in Table 5.

**TABLE 5:** Research summary of artificial intelligence in age-related macular degeneration.

Year	Country or region	Authors	Task	Dataset (disease images)	AI algorithm	Output
2022	Korea	Han et al. (2022)	Diagnosis	4,749 images (2,624 images)	VGG-16, VGG-19, ResNet	Accuracy = 0.874
2021	America	Tak et al. (2021)	Classification	420 images (420 images)	Convolutional neural networks	Accuracy = 0.88
2021	Taiwan	Chou et al. (2021)	Diagnosis	699 images (491 images)	EfficientNet-B3	Accuracy = 0.8367, Sensitivity = 0.8076, Specificity = 0.8472, AUC = 0.8857
2020	Korea	Heo et al. (2020)	Diagnosis	399 images (399 images)	VGG16	Accuracy = 0.9086
2022	America	Ganjdanesh et al. (2022)	Prediction	30,000 images (30,000 images)	ResNet-18	Accuracy = 0.905, AUC = 0.762
2022	China	Song et al. (2022)	Prediction	671 images (671 images)	Classified convolution neural network, complete convolution neural network	Accuracy = 0.930, Dice coefficients = 0.873, Sensitivity = 0.873, Specificity = 0.922
2022	Taiwan	Yeh et al. (2022)	Prediction	698 images (698 images)	Deep convolution neural network	AUC = 0.989, Accuracy = 0.936, Sensitivity = 0.933, Specificity = 0.938
2020	America	Yan et al. (2020)	Prediction	31,262 images, 52 related mutated genes (31,262 images)	Convolutional neural networks	AUC = 0.85
2022	Austria	Holomcik et al. (2022)	Division	9,268 images (9,268 images)	U-Net	F1 score = 0.65, Accuracy = 0.75, Recall = 0.72
2022	China	He et al. (2022)	Detection	UCSD dataset, Duke dataset (46,421 images)	ResNet-50, Local outlier factor	UCSD: Accuracy = 0.9987
						Duke: Accuracy = 0.9756

## 4 Limitations and challenges

Based on the referenced studies, AI is widely used in retinal vascular diseases, especially in image recognition and data analysis. Although AI model shows superior performance in assisting the diagnosis, identification, screening, staging and grading of retinal vascular diseases, AI model also faces many limitations and challenges in the research process, which will seriously affect the further research of artificial intelligence in retinal vascular diseases and hinder its clinical application. Below, we list the main limitations and challenges of AI in research on retinal vascular diseases. 1) Image quality in the dataset (Aronson, 2022; Gutierrez et al., 2022): The image quality used in AI research has a significant impact on AI research. The higher the image quality, the better the performance of the AI model. However, the quality of the image is related to a variety of factors, such as shooting equipment, operators, the degree of cooperation of patients and so on. Therefore, high-quality images should be used as much as possible in AI research. 2) Manual annotations of images in the dataset (Hashimoto et al., 2020; Betzler et al., 2022): The images in many studies must be manually annotated, and the accuracy of manual labeling has a significant impact on the performance of the AI model. This requires experts in related diseases to label the images to ensure the validity of the data. 3) Sample size of the dataset (Ji et al., 2022b): The accuracy of the AI model is related to the sample size. The larger the sample size, the higher the accuracy of the AI model. The sample size of the dataset used in some studies was small, which had an impact on the performance of the AI model. Therefore, in the study, the sample size of the dataset should be expanded as much as possible to ensure the accuracy of the AI model. 4) Patient heterogeneity (Galante et al., 2023): Studies on the AI model are likely to be affected by different patient groups. Differences between patients such as age, sex, race, and region affect the performance of the AI model. If only one patient group is included in the data set used in the study, it will seriously affect the accuracy and clinical application of the AI model. 5) Clinical application of the AI model (Al-Aswad et al., 2022; Wawer Matos et al., 2022): Although in many studies, AI model shows superior performance in external verification datasets, due to the great difference between “real environment” and “research environment”, this will lead to a series of problems in clinical application of AI model, which will affect the performance of AI model. 6) Clinicians' reserve of AI algorithms and their related knowledge (Tabuchi, 2022; Yang et al., 2023): AI belongs to a branch of computer science and does not belong to the professional scope of clinicians, which leads to clinicians' lack of knowledge about AI algorithms, their related knowledge, and lack of explanation, which can easily lead to the “black box phenomenon” and hinder the application of AI in clinical work.

## 5 Conclusion

At present, the use of AI technology to assist clinicians in the study of ophthalmic images and other ophthalmic examinations is a current major focus. The combination of AI and ophthalmology will greatly improve the diagnosis of ophthalmic diseases, especially retinal vascular diseases based on the analysis of fundus images. The diagnosis model based on AI will be beneficial for the early detection, diagnosis, and treatment of retinal vascular diseases. Although the application of artificial intelligence in the field of ophthalmology has made a lot of research results, but from the overall situation, it is only the beginning. With further developments in computer science and technology, the application of AI in the field of ophthalmology will be more and more widely used in the field of ophthalmology. In addition, with the deepening of research, in addition to image processing and recognition, other artificial intelligence technologies will also carry out related research in the field of ophthalmology, so as to promote the continuous development of ophthalmology.
